# Neoadjuvant chemotherapy or primary surgery for stage III/IV ovarian cancer: contribution of diagnostic laparoscopy

**DOI:** 10.1186/1471-2407-9-171

**Published:** 2009-06-06

**Authors:** Jean-Luc Brun, Roman Rouzier, Frédéric Selle, Sidney Houry, Serge Uzan, Emile Daraï

**Affiliations:** 1 AP-HP, Hôpital Tenon, Department of Gynecology, 4 rue de la Chine, F-75571 Paris Cedex 12, France; 2 AP-HP, Hôpital Tenon, Department of Oncology, 4 rue de la Chine, F-75571 Paris Cedex 12, France; 3 AP-HP, Hôpital Tenon, Department of Surgery, 4 rue de la Chine, F-75571 Paris Cedex 12, France; 4 UPMC University Paris 06, UPRES EA 4053, F-75005, Paris, France

## Abstract

**Background:**

The aims of this retrospective study were to evaluate laparoscopic triage of patients with advanced ovarian cancer towards primary surgery or neoadjuvant chemotherapy, and to analyze outcome according to the treatment.

**Methods:**

Between January 2001 and December 2006, 55 patients with stage III – IV ovarian cancer underwent diagnostic laparoscopy. Primary surgery was performed when complete cytoreduction was considered feasible, while the other patients received neoadjuvant chemotherapy (platinum-based combination with taxanes) and interval surgery. All the patients received adjuvant chemotherapy.

**Results:**

Patients treated with neoadjuvant chemotherapy (n = 29) had a higher mean body mass index (P = 0.048), higher serum CA 125 levels (P = 0.026), and more metastases (P = 0.045) than patients treated with primary surgery (n = 26). In patients treated with primary surgery, complete cytoreduction and a residual tumour size ≤ 2 cm were obtained in respectively 54% and 77% of cases. Complete cytoreduction was achieved in respectively 100% and 33% of cases when primary surgery was performed by an oncologic gynaecologist and by a non-oncologic gynaecologist (P = 0.002). Interval surgery yielded complete cytoreduction and a residual tumour size ≤ 2 cm in respectively 73% and 85% of cases. With a median follow-up of 24 months (range 7 – 78 months), the survival rates after primary surgery and interval surgery were 61% and 66% respectively.

**Conclusion:**

Diagnostic laparoscopy is useful for identifying patients with stage III/IV ovarian cancer who qualify for primary cytoreduction. Surgeon experience was a determining factor for the success of complete cytoreduction.

## Background

The extent of cytoreductive surgery and the amount of residual tumour are the most important survival determinants in patients with advanced ovarian cancer [1,2]. In order to increase the rate of complete or optimal debulking and to limit peri-operative morbidity, neoadjuvant chemotherapy with interval cytoreduction has emerged as an alternative to primary surgery. This delayed strategy does not seem to compromise survival [3,4]. However, although neoadjuvant chemotherapy is an acceptable alternative for patients with unresectable disease, Bristow et al have stressed that survival appears to be poorer after initial chemotherapy than after successful up-front cytoreductive surgery [5]. Hence, the main issue is how to evaluate the resectability of stage III/IV ovarian cancer.

Despite improvements in CT, MR and PET scan, and in tumour markers, the resectability of intraperitoneal disease remains difficult to judge [6-8]. Several predictive models have been proposed, based on clinical findings (ascites, etc.), imaging, and the CA125 serum level, but false-positive rates range from 5% to 37% and surgical evaluation is therefore crucial [6-8].

Diagnostic laparoscopy was first proposed as a second-look procedure for guiding subsequent treatment, but it proved to be less reliable than laparotomy [9]. Diagnostic laparoscopy was then used to assess the resectability of advanced ovarian cancer, with a view to either primary cytoreductive surgery or neoadjuvant chemotherapy without impacting on its delay [10]. In patients whose disease appears resectable on laparoscopy, optimal debulking rates range from 67% to 96% [10-15].

The aims of this longitudinal retrospective study were to examine whether, in patients with stage III/IV ovarian cancer, diagnostic laparoscopy can select those with a high likelihood of complete cytoreduction, and to compare the operative procedures, recurrence rates and survival rates with those in patients receiving neoadjuvant chemotherapy.

## Methods

### Patients

Between January 2001 and December 2006, 72 patients with strong evidence of advanced ovarian cancer (FIGO stage III – IV) underwent surgical exploration in our department. The inclusion criteria were good nutritional status, and no contraindication to surgery.

Preoperative evaluation included general and gynaecological examination, pelvic sonography, serum CA 125 assay, routine blood tests, EKG, chest radiography and computed abdominopelvic tomography (CT).

Fifty-five patients had diagnostic laparoscopy to assess the possibility of complete debulking surgery (defined as no visible residual tumour). Laparoscopy has been progressively introduced since 2001 and is now considered as a standard of care in our institution. Patients were informed that surgery included a preliminary laparoscopic approach to assess resectability. Therefore, informed consent was obtained for the whole surgery itself.

### Surgical technique

A longitudinal midline incision of approximately 15 mm was made just above or below the umbilicus. The rectus sheath was identified and incised longitudinally. The peritoneum was opened and a blunt 10-mm disposable trocar was gently introduced into the peritoneal cavity under visual and digital control. A second 5-mm trocar was then inserted under direct visual control into the midline, about 40 to 50 mm above the pubic arch. An attempt was made to inspect the entire abdominal cavity systematically, by using atraumatic forceps: the ovaries, fallopian tubes, uterus, pelvic peritoneum, serosa and mesentery of the large and small bowel, liver surface, paracolic gutters and diaphragm were carefully examined. When necessary, an additional 5-mm trocar was used to facilitate dissection of adhesions with scissors and bowel manipulation. Biopsy specimens of the ovaries or metastatic nodules or peritoneal surface were used for extemporaneous frozen section to confirm the diagnosis of ovarian cancer. At the end of the laparoscopic procedure, all the layers of incised abdomen were closed separately with resorbable sutures at level of the umbilical trocar site, to avoid trocar metastases.

The procedures were performed by seven surgeons with adequate laparoscopic training; three were experts in gynaecologic oncology and four were non-oncologic gynaecologic surgeons. Twenty-six patients were treated by the gynaecologic oncologists (47%).

### Study design

After complete laparoscopic exploration of the pelvis and abdomen, the patients either underwent laparotomy and primary cytoreductive surgery through a midline xifo-pubic incision, or received neoadjuvant chemotherapy followed by interval debulking surgery and subsequent adjuvant chemotherapy.

Patients had primary cytoreductive surgery only when complete macroscopic cytoreduction (absence of visible residual tumour) was considered feasible. Primary debulking surgery included total hysterectomy, bilateral salpingo-oophorectomy, total infragastric omentectomy, appendectomy, peritonectomy (limited to the pelvis, the paracolic gutters, or focally in the anterolateral diaphragmatic area), spleen resection when necessary, and rectosigmoid resection when indicated. Pelvic, common iliac, and infrarenal paraaortic lymphadenectomy was part of the standard operation for patients with good medical status at the end of debulking surgery and with complete macroscopic cytoreduction. Adjuvant chemotherapy always consisted of carboplatin and paclitaxel administered every 3 weeks. Six chemotherapy cycles were planned.

Neoadjuvant chemotherapy was indicated when the surgeon judged that optimal cytoreduction could not be achieved. The reasons for choosing neoadjuvant chemotherapy instead of primary debulking surgery included factors related to the patient herself (comorbidity, increased anaesthetic risk) and to the extent of the disease (findings during diagnostic open laparoscopy). Surgical findings influencing the decision to opt for neoadjuvant chemotherapy included extensive visceral peritoneal disease (diffuse superficial involvement of organs such as the small bowel, mesentery, large bowel, liver and gallbladder), and extensive involvement of the upper abdomen (diaphragm and liver, or hepatic pedicle).

Neoadjuvant chemotherapy always consisted of a platinum-based combination with taxanes, administered every 3 weeks. The number of chemotherapy cycles depended on the response, based on clinical examination, serum CA 125 assay and CT scan. The patients were then referred for standard debulking, as described above. The size of the residual tumour at the end of surgery was recorded. After debulking, chemotherapy combining carboplatin and paclitaxel was subsequently administered to all patients.

No attempt was made to compare the predictive value of imaging methods and laparoscopy.

Demographics, the disease stage and the histological type were first compared between the neoadjuvant chemotherapy and primary surgery groups. Categorical variables were compared with Fisher's exact test or the chi-square test, depending on the sample size. Quantitative data were compared with the Mann-Whitney test. The surgical response, recurrence rate and survival were then compared. Disease-free survival curves after diagnosis (first surgical procedure) were calculated according to the Kaplan-Meier method, and compared with the log-rank test. Significance was assumed at a P value < 0.05.

## Results

### Characteristics of the patients undergoing primary surgery and neoadjuvant chemotherapy

Among the 55 patients referred to our department for laparoscopic evaluation of stage III – IV ovarian cancer, 26 patients underwent primary surgery after diagnostic laparoscopy. Histological diagnosis was based on the salpingo-oophorectomy specimen in all 26 patients.

Twenty-nine patients received neoadjuvant chemotherapy, because of severe comorbidity in 3 cases and extensive disease in 26 cases. Suboptimal resection was predicted on the basis of extensive peritoneal disease involving the upper abdomen and mesentery and/or requiring multiple bowel resection in all 26 patients. The histological diagnosis was based on peritoneal biopsy in 17 patients (59%), ovarian biopsy in 8 (27%), salpingo-oophorectomy in 3 (10%), and partial omentectomy in 1 (3%). The three patients with severe comorbidity died one month after the first cycle of neoadjuvant chemotherapy and were excluded from the study population because interval surgery could not be performed. The causes of death were not related to the toxicity of the chemotherapy but to bowel obstruction in two patients and pulmonary embolism in one patient.

The characteristics of the 52 remaining patients are summarized in Table 1. Patients treated with neoadjuvant chemotherapy had a higher body mass index (BMI), a higher serum CA 125 level, and more metastases than patients treated with primary surgery.

**Table 1:** Characteristics of the patients

Characteristic	Neoadjuvant chemotherapy (n = 26)	Primary surgery (n = 26)	*P*
Median age (y, range)	62 (21 – 88)	60 (26 – 81)	*NS*
Median parity (range)	1 (0 – 7)	1 (0 – 4)	*NS*
Median BMI * (range)	24 (19 – 31)	22 (16 – 30)	*0.048*
BMI ≥ 30 (%)	2 (8)	2 (8)	*NS*
Post-menopausal (%)	21 (81)	22 (85)	*NS*
Cancer history ** (%)			
Familial	6 (23)	10 (38)	*NS*
Personal	0	2 (8)	
Familial and personal	2 (8)	1 (4)	
None	18 (69)	13 (50)	
Stage (%)			
IIIB	1 (4)	3 (12)	*0.045*
IIIC	20 (77)	23 (88)	
IV	5 (19)	0	
Histology (%)			
Serous	16 (61)	14 (54)	*NS*
Mucinous	1 (4)	0	
Endometrioid	6 (23)	7 (27)	
Clear cells	0	4 (15)	
Undifferentiated	2 (8)	1 (4)	
Other	1 (4)	0	
Grade (%)			
1	2 (8)	4 (16)	*NS*
2	13 (50)	11 (42)	
3	9 (34)	11 (42)	
Unknown	2 (8)	0	
Emboli (%)	6 (23)	7 (27)	*NS*
Pleural effusion (%)	3 (12)	0	*NS*
Pre-operative CA125 (%)			
≤ 500 U/ml	8 (31)	16 (61)	*0.026*
> 500 U/ml	18 (69)	10 (39)	

* BMI: Body mass index; ** Only breast, uterus, ovarian and colon cancer were considered.

### Results of primary surgery after diagnostic laparoscopy (table 2)

**Table 2:** Results of surgery (no significant differences)

Characteristic	Neoadjuvant chemotherapy (n = 26)	Primary surgery (n = 26)
Total hysterectomy (%)	22 (85)	23 (88)
Bilateral salpingo-oophorectomy (%)	25 (96)	26 (100)
Omentectomy (%)	24 (92)	24 (92)
Rectosigmoid resection (%)	5 (19)	8 (31)
Multiple bowel resection (%)	1 (4)	1 (4)
Cytology of ascites (%)		
Positive	8 (42)	14 (54)
Negative	11 (58)	12 (46)
ND	7	0
Lymphadenectomies (%)	13 (50)	17 (65)
Median lymph nodes removed (range)	25 (10 – 50)	23 (4 – 50)
Median lymph nodes involved (range)	1 (0 – 4)	2 (0 – 14)
Median ratio involved/removed (range)	4 (0 – 36)	7 (0 – 57)
Complete cytoreduction	19 (73)	14 (54)
Residual tumour (%)		
≤ 1 cm	2 (8)	4 (15)
1.1 – 2 cm	1 (4)	2 (8)
> 2 cm	4 (15)	6 (23)
Location of residual tumour* (%)		
Pelvis	2 (15)	3 (19)
Upper abdomen	6 (46)	6 (37.5)
Peritoneal, extensive	4 (31)	6 (37.5)
Retroperitoneal	1 (8)	1 (6)

* Some patients had more than one location.

Residual tumours smaller than 2 cm (optimal surgery) were obtained in 20 cases (77%). Fourteen patients (54%) had no macroscopic residue (i.e. complete cytoreduction) after primary surgery. The surgeon's expertise influenced the probability of complete cytoreduction: cytoreduction was complete in 100% of cases when debulking surgery was performed by an oncologic gynaecologist, compared to only 33% of cases when performed by a non-oncologic gynaecologist (P = 0.002). Primary surgery was combined with lymphadenectomy in 17 patients with residual tumours ≤ 1 cm.

The adjuvant chemotherapy regimen was carboplatin and paclitaxel in all 26 patients. The median number of adjuvant chemotherapy cycles was 6 (range, 1–9). Five patients died before completing the 6 cycles. Three patients with incomplete response to chemotherapy (abnormal CA 125 levels at the end of the 6 cycles) underwent 3 additional cycles of the same regimen.

In one patient treated with primary surgery, a metastasis occurred 5 months after the procedure at the insertion site of the ancillary trocar. This patient had a residual tumour > 2 cm after surgery and was considered chemoresistant.

### Surgical treatment of patients receiving neoadjuvant chemotherapy

The median interval between diagnostic laparoscopy and the beginning of neoadjuvant chemotherapy was 11 days (range: 1–30). The neoadjuvant chemotherapy regimens were carboplatin-paclitaxel for 23 patients (88%), carboplatin-docetaxel for 1 patient (4%), and oxaliplatin-docetaxel for 2 patients (8%). The median number of neoadjuvant chemotherapy cycles was 4 (range, 3–8). Fifteen patients (58%) received 3 or 4 cycles of neoadjuvant chemotherapy. All 15 of these patients responded after 3 cycles, based on clinical examination, the serum CA 125 level, and abdominopelvic CT. Eleven patients (42%) who did not respond after 3 cycles received 6 cycles or more. All 11 of these patients underwent interval surgery, when the disease was considered stable (6 patients) or in regression (5 patients).

The response to neoadjuvant chemotherapy was judged from the tumour burden at interval surgery. In 17 patients (65%) the largest tumour was smaller than 2 cm after neoadjuvant chemotherapy. Twelve of these patients had required only 3 or 4 cycles of neoadjuvant chemotherapy. No macroscopic tumour residue was found in 6 cases (after 3 cycles in 4 cases). The remaining 9 patients (35%) had tumours ranging from 3 to 13 cm after 3 cycles (n = 3) or more than 6 cycles (n = 6) of neoadjuvant chemotherapy.

The results of interval surgery are summarized in table 2. Residual tumours of less than 2 cm were obtained in 22 cases (85%). Complete cytoreduction was obtained in 19 cases (73%). The surgeon's expertise did not influence the probability of complete cytoreduction; cytoreduction was complete in 60% and 81% of cases when debulking surgery was performed by an oncologic gynaecologist and a non-oncologic gynaecologist, respectively.

No trocar metastases occurred in patients treated with neoadjuvant chemotherapy.

After debulking, chemotherapy combining carboplatin and paclitaxel was administered to 20 patients until 6 cycles. For 5 patients with incomplete response to 6 cycles of carboplatin paclitaxel, a second line chemotherapy with gemcitabin or topotecan was administered. The remaining patient died post-operatively from the evolution of the disease before receiving the first post-operative cycle of chemotherapy.

The median number of chemotherapy cycles was 8 (range, 4–16). The median number of post-operative chemotherapy cycles was 4 (range, 2–8).

### Survival after primary and interval surgery

Survival probabilities were calculated according to the treatment strategy. The median follow-up was 24 months (range 7 – 78). Including the three patients who died before interval surgery, the survival rates were 66% and 61%, respectively, among patients who had neoadjuvant chemotherapy and primary surgery.

**Figure 1:**
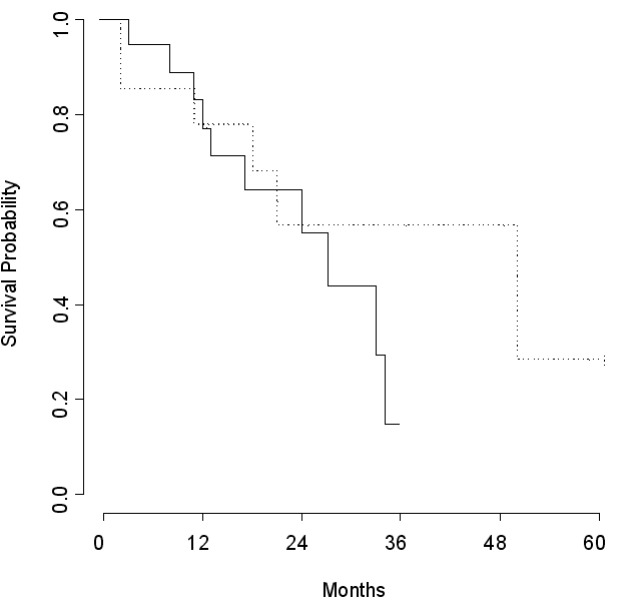
Survival probability among patients with no or microscopic residual tumour after primary (---) and interval (—) surgery (P = 0.28).

In patients with complete cytoreduction (no or microscopic residual tumours), the respective median disease-free survival times after primary surgery and after neoadjuvant chemotherapy followed by interval surgery were 50 months and 27 months (Figure 1). In patients with no residual disease, the median disease-free survival times were 50 months and 33 months respectively (P = 0.04). In patients with macroscopic residual tumours, the median disease-free survival times after primary surgery and after neoadjuvant chemotherapy followed by interval surgery were 25 months and 22 months, respectively. There was no significant difference in disease-free survival between patients who had primary surgery and those who had interval surgery, except in the subgroup of patients with no macroscopic and microscopic residual tumours.

## Discussion

This study shows that diagnostic laparoscopy can reliably identify patients with advanced-stage ovarian cancer who are likely to have optimal cytoreduction during primary surgery. When diagnostic laparoscopy showed unresectable disease, the rate of optimal cytoreduction during interval surgery following neoadjuvant chemotherapy was similar to that achieved during primary surgery in patients with resectable disease. However, survival tended to be better after complete cytoreduction during primary surgery than during interval surgery.

Half of the patients, all of whom had stage III ovarian cancer, were considered to be good candidates for primary cytoreductive surgery on the basis of diagnostic laparoscopy. These results are in keeping with previous studies underlying the contribution of laparoscopy to evaluate resectability [12,15]. Using extensive visceral peritoneal metastases, extensive involvement of the upper abdomen, extensive small bowel involvement and multiple liver metastases as criteria of unresectability, Angioli et al reported that 61% of patients were considered operable on the basis of diagnostic laparoscopy [12]. In our series, 54% of patients who were considered operable on the basis of diagnostic laparoscopy had complete macroscopic cytoreduction, and respectively 69% and 77% of patients had optimal cytoreduction defined by a residual tumour size of ≤ 1 cm and ≤ 2 cm. These results are better than those of previous laparotomic studies, in which optimal cytoreduction (< 2 cm residual tumour) was achieved in 35% to 50% of patients [4,14]. The use of diagnostic laparoscopy to identify candidates for primary cytoreductive surgery among patients with advanced-stage ovarian cancer is controversial. In a multicenter study of 77 patients with stage III-IV ovarian cancer, Vergote et al reported that diagnostic laparoscopy selected 28 patients for primary surgery, and that cytoreductive surgery was optimal (< 0.5 cm largest residual tumour size) in 79% of these 28 patients [10]. These results may appear better than ours, but it should be noted that, contrary to us, the latter authors used clinical and radiological findings to select patients for laparoscopy. Angioli et al [12] reported complete cytoreductive surgery (no residual tumour) in 96% of patients whose diagnostic laparoscopy showed tumor resectability, while Fagotti et al reported optimal cytoreduction (residual tumour ≤ 1 cm) in 67% of such patients [15].

In our study, the surgeons with expertise in gynaecologic oncology achieved complete cytoreduction during primary surgery in 100% of cases. In previous reports, optimal cytoreduction during primary surgery was achieved in 20% to 90% of cases, according to the experience of the surgeon and the centre [16-19]. Although the definition of optimal cytoreduction remains debatable, it should be noted that the cut-off of 1 cm residual disease commonly used does not reflect major differences in terms of prognosis. Indeed, in a retrospective review of 1895 patients with stage III ovarian cancer carried out by the Gynecologic Oncology Group (GOG), patients with 0.1 to 1.0 cm and > 1.0 cm residual disease had a 2 – 2.5 fold increased risk of recurrence and death compared with patients with no macroscopic residual disease [20].

The main issue in advanced-stage ovarian cancer is how to select candidates for primary cytoreductive surgery and therefore to avoid unnecessary laparotomy with suboptimal cytoreduction that would delay chemotherapy. Given the re-growth of tumour cells between chemotherapy cycles, cytoreductive surgery should be maximally beneficial in patients with complete cytoreduction or limited tumour residues [21,22]. Previous studies have suggested that voluminous ascites was associated with suboptimal cytoreductive surgery [23,24]. Bristow et al proposed a score based on radiological variables such as peritoneal thickening, peritoneal implants > 2 cm, bowel mesentery tumours > 2 cm, suprarenal lymph nodes > 1 cm, omental extension to the spleen, pelvic sidewall involvement, and hydroureter to identify unresectable disease [7]. Models based on clinical and CT findings as well as serum tumour markers have also been developed [5], but their false-negative rates range from 5% to 37% [6-9]. Moreover, cross-validation of these predictive algorithms also failed to identify good candidates for neoadjuvant chemotherapy. Indeed, 62% to 86% of patients considered to have had suboptimal surgery had optimal cytoreduction [5]. In contrast, diagnostic laparoscopy can accurately identify patients who may benefit from primary complete cytoreduction. A laparoscopy-based score for predicting surgical outcome was recently developed by Fagotti et al on the basis of a pilot study [15]. Omental cake, peritoneal carcinomatosis, diaphragmatic carcinomatosis, mesenteric retraction, bowel and/or stomach infiltration, and liver metastases were each assigned a value of 2. In the final model, a predictive index score of ≥ 8 had a specificity of 100%, a positive predictive value of 100% and a negative predictive value of 70% for prospectively identifying patients with residual tumours after debulking surgery. External validation by other teams is required to confirm the usefulness of diagnostic laparoscopy in this setting, and to develop a scoring system to predict resectability.

Interval surgery allowed complete cytoreduction in 73% of our patients with unresectable disease at diagnostic laparoscopy. Our rate of optimal cytoreductive surgery is in keeping with that reported elsewhere with neoadjuvant chemotherapy (62% to 94%) [12,25,26]. Likewise, Angioli et al reported complete debulking in 80% of cases [12], although their intention-to-treat data were not reported. After reintegration of nine patients excluded from interval surgery, the rate of complete cytoreduction during interval surgery fell to 59%. In our study, the frequency of intestinal resection was the same in patients undergoing primary surgery as in those undergoing interval surgery with neoadjuvant chemotherapy. In the same way, the number of positive lymph nodes and the ratio of positive to total nodes did not differ between the two groups, suggesting that neoadjuvant chemotherapy was not effective on lymph node involvement. Although Morice et al found no difference in overall and disease-free survival between patients undergoing primary cytoreductive surgery and those receiving neoadjuvant chemotherapy and cytoreductive interval surgery, interval surgery was associated with less morbidity [26]. Likewise, Chan et al found that their patients' overall quality of life and functional status improved after neoadjuvant chemotherapy [27]. However, in a recent meta-analysis, Bristow et al found that neoadjuvant chemotherapy was associated with poorer survival relative to initial surgery [5]. This can be explained by the inclusion of non-randomized studies in this meta-analysis [5]. Moreover, patients receiving the neoadjuvant chemotherapy had clinically worse disease than those chosen to undergo primary surgery and should have been expected to have worse survival. Although no significant difference was observed in our study, probably owing to a small sample size and short follow-up, survival tended to be better after primary surgery than after interval surgery among women who had complete cytoreduction. Probably due to the same limits of the study, no such difference was observed between the patients with residual tumours after primary and interval surgery. However, in the meta-analysis of Bristow et al, patients who were referred for neoadjuvant chemotherapy were considered unresectable solely on the basis of physical examination, CT findings and/or serum marker levels, possibly excluding some patients who would have qualified for potentially complete primary cytoreduction [5].

## Conclusion

Our results support the use of diagnostic laparoscopy for identifying patients with advanced-stage ovarian cancer who are likely to benefit from primary surgery with complete cytoreduction, and the importance of the primary surgery being performed by an oncologic gynaecologist. Pending the results of the EORTC trial comparing neoadjuvant chemotherapy with primary cytoreduction (EORTC 55971), laparoscopy should be used to identify patients qualifying for complete primary cytoreductive surgery.

## Competing interests

The authors declare that they have no competing interests.

## Authors' contributions

J-LB contributed to the collection, analysis and interpretation of the data, as well as drafting the manuscript. RR performed the statistical analysis and was involved in drafting the manuscript. FS provided clinical records from the patients of the Department of Oncology and critically revised the manuscript. SH provided clinical records from the patients of the Department of Surgery and critically revised the manuscript. SU was responsible for the Department of Gynaecology and was involved in revising the content of the manuscript. ED initiated and coordinated the study and drafted the manuscript. All authors read and approved the final manuscript.

## Pre-publication history

The pre-publication history for this paper can be accessed here:

http://www.biomedcentral.com/1471-2407/9/171/prepub

## References

[B1] Griffiths CT Surgical resection of tumor bulk in the primary treatment of ovarian carcinoma Natl Cancer Inst Monogr 1975 42 101 4 1234624

[B2] Chi DS Liao JB Leon LF Venkatraman ES Hensley ML Bhaskaran D Hoskins WJ Identification of prognostic factors in advanced epithelial ovarian carcinoma Gynecol Oncol 2001 82 532 7 10.1006/gyno.2001.6328 11520151

[B3] Baekelandt M The potential role of neoadjuvant chemotherapy in advanced ovarian cancer Int J Gynecol Cancer 2003 13 163 8 10.1111/j.1525-1438.2003.13354.x 14656274

[B4] Huober J Meyer A Wagner U Wallweiner D The role of neoadjuvant chemotherapy and interval laparotomy in advanced ovarian cancer J Cancer Res Clin Oncol 2002 128 153 60 10.1007/s00432-001-0312-3 11935302 PMC12164442

[B5] Bristow RE Eisenhauer EL Santillan A Chi DS Delaying the primary surgical effort for advances ovarian cancer: a systematic review of neoadjuvant chemotherapy and interval cytoreduction Gynecol Oncol 2007 104 480 90 10.1016/j.ygyno.2006.11.002 17166564

[B6] Martinez-Said H Rincon DG Montes De Oca MM Ruiz GC Ponce JLA Lopez-Graniel CM Predictive factors for irresectability in advanced ovarian cancer Int J Gynecol Cancer 2004 14 423 30 10.1111/j.1048-891x.2004.014301.x 15228414

[B7] Bristow RE Duska L Lambrou N Fishman EK O'Neill MJ Trimble EL Montz FJ A model for predicting surgical outcome in patients with advanced ovarian carcinoma using computed tomography Cancer 2000 89 1532 40 10.1002/1097-0142(20001001)89:7<1532::AID-CNCR17>3.0.CO;2-A 11013368

[B8] Dowdy S Mullany S Brandt K Huppert B Cliby W The utility of computed tomography scans in predicting suboptimal cytoreductive surgery in women with advanced ovarian carcinoma Cancer 2004 101 346 52 10.1002/cncr.20376 15241833

[B9] Clough KB Ladonne JM Nos C Renolleau C Validire P Durand JC Second look for ovarian cancer: laparoscopy or laparotomy? A prospective comparative study Gynecol Oncol 1999 72 411 7 10.1006/gyno.1998.5272 10053115

[B10] Vergote I De Wever I Tjalma W Van Gramberen M Decloedt J van Dam P Neoadjuvant chemotherapy or primary debulking surgery in advanced ovarian carcinoma: a retrospective analysis of 285 patients Gynecol Oncol 1998 71 431 6 10.1006/gyno.1998.5213 9887245

[B11] Fagotti A Fanfani F Ludovisi M Lo Voi R Bifulco G Testa AC Scambia G Role of laparoscopy to assess the chance of optimal cytoreductive surgery in advanced ovarian cancer: a pilot study Gynecol Oncol 2005 96 729 35 10.1016/j.ygyno.2004.11.031 15721418

[B12] Angioli R Palaia I Zullo MA Muzii L Manci N Calcagno M Benedetti Panici P Diagnostic open laparoscopy in the management of advanced ovarian cancer Gynecol Oncol 2006 100 45 61 10.1016/j.ygyno.2005.09.060 16325244

[B13] Deffieux X Castaigne D Pomel C Role of laparoscopy to evaluate candidates for complete cytoreduction in advanced stages of epithelial ovarian cancer Int J Gynecol Cancer 2006 16 Suppl 1 35 40 10.1111/j.1525-1438.2006.00323.x 16515565

[B14] Bristow RE Tomacruz RS Armstrong DK Trimble EL Monz FJ Survival effect of maximal cytoreductive surgery for advanced ovarian carcinoma during platinum era: a meta-analysis J Clin Oncol 2002 20 1248 59 10.1200/JCO.20.5.1248 11870167

[B15] Fagotti A Ferrandina G Fanfani F Ercoli A Lorusso D Rossi M Scambia G A laparoscopy-based score to predict surgical outcome in patients with advanced ovarian carcinoma: a pilot study Ann Surg Oncol 2006 13 1156 61 10.1245/ASO.2006.08.021 16791447

[B16] Unzelman RF Advanced epithelial ovarian carcinoma: long-term survival experience at the community hospital Am J Obstet Gynecol 1992 166 1663 71 1615973 10.1016/0002-9378(92)91554-n

[B17] Junor EJ Hole DJ Gillis CR Management of ovarian cancer: referral to a multidisciplinary team matters Br J Cancer 1994 70 363 70 8054286 10.1038/bjc.1994.307 PMC2033481

[B18] Eisenkop SM Spirtos NM Montag TW Nalick RH Wang HJ The impact of subspecialty training on the management of advanced ovarian cancer Gynecol Oncol 1992 47 203 9 10.1016/0090-8258(92)90107-T 1468698

[B19] Michel G DeIaco P Castaigne D el-Hassan MJ Lobreglio R Lhomme C Rey A Duvillard P Extensive cytoreductive surgery in advanced ovarian carcinoma Eur J Gynaecol Oncol 1997 18 9 15 9061314

[B20] Winter WE Maxwell GL Tian C Carlson JW Ozols RF Rose PG Markman M Armstrong DK Muggia F McGuire WP Prognostic Factors for Stage III Epithelial Ovarian Cancer: A Gynecologic Oncology Group Study J Clin Oncol 2007 25 3621 27 10.1200/JCO.2006.10.2517 17704411

[B21] Tannock I Cell kinetics and chemotherapy: a critical review Cancer Treat Rep 1979 62 1117 33 356975

[B22] Golde JH Coldman AJ A mathematical model for relating the drug sensitivity of tumors to the spontaneous mutation rate Cancer Treat Rep 1979 63 1727 33 526911

[B23] Kuhn W Rutke S Spathe K Schmalfeldt B Florack G vonHundelshauen B Pachyn D Ulm K Graeff H Neoadjuvant chemotherapy followed by tumor debulking prolongs survival for patients with poor prognosis in International Federation of Gynecology and Obstetrics stage IIIC ovarian carcinoma Cancer 2001 92 2581 91 10.1002/1097-0142(20011115)92:10<2585::AID-CNCR1611>3.0.CO;2-# 11745193

[B24] Dowdy S Mullany S Brandt K Huppert B Cliby W The utility of computed tomography scans in predicting suboptimal cytoreductive surgery in women with advanced ovarian carcinoma Cancer 2004 101 346 52 10.1002/cncr.20376 15241833

[B25] Inciura A Simavicius A Juozaityte E Kurtinaitis J Nadisauskiene R Svedas E Kajenas S Comparison of adjuvant and neoadjuvant chemotherapy in the management of advanced ovarian cancer: a retrospective study of 574 patients BMC Cancer 2006 6 153 16759398 10.1186/1471-2407-6-153 PMC1533845

[B26] Morice P Dubernard G Rey A Atallah D Pautier P Pomel C Lhommé C Duvillard P Castaigne D Results of interval debulking surgery compared with primary debulking surgery in advanced stage ovarian cancer J Am Coll Surg 2003 197 955 63 10.1016/j.jamcollsurg.2003.06.004 14644284

[B27] Chan YM Ng TY Ngan HYS Wong LC Quality of life in women treated with neoadjuvant chemotherapy for advanced ovarian cancer: a prospective longitudinal study Gynecol Oncol 2003 88 9 16 10.1006/gyno.2002.6849 12504620

